# Mechanism and Complex Roles of HSC70 in Viral Infections

**DOI:** 10.3389/fmicb.2020.01577

**Published:** 2020-07-21

**Authors:** Zeng Wang, Yongtao Li, Xia Yang, Jun Zhao, Yuening Cheng, Jianke Wang

**Affiliations:** ^1^College of Animal Science and Veterinary Medicine, Henan Agricultural University, Zhengzhou, China; ^2^Institute of Special Animal and Plant Sciences, Chinese Academy of Agricultural Sciences, Changchun, China; ^3^Department of Microbiology, Molecular Genetics and Immunology, University of Kansas Medical Center, Kansas City, KS, United States

**Keywords:** HSC70, virus, infection, regulatory mechanism, role

## Abstract

Heat shock cognate 71-kDa protein (HSC70), a constitutively expressed molecular chaperon within the heat shock protein 70 family, plays crucial roles in maintaining cellular environmental homeostasis through implicating in a wide variety of physiological processes, such as ATP metabolism, protein folding and transporting, antigen processing and presentation, endocytosis, and autophagy. Notably, HSC70 also participates in multiple non-communicable diseases and some pathogen-caused infectious diseases. It is known that virus is an obligatory intracellular parasite and heavily relies on host machineries to self-replication. Undoubtedly, HSC70 is a striking target manipulated by virus to ensure the successful propagation. In this review, we summarize the recent advances of the regulatory mechanisms of HSC70 during viral infections, which will be conducive to further study viral pathogenesis.

## Introduction

**FIGURE 1:**
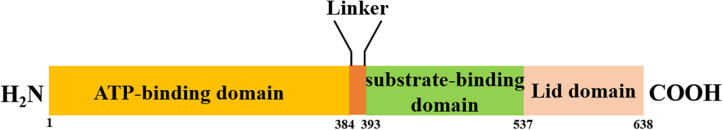
Schematic representation of HSP70s structure. All chaperons in the HSP70 family encompass three main structural domains, namely, an N-terminal ATP-binding domain (NBD) that exhibits ATPase activity, a substrate-binding domain (SBD) to interact with substrates, and a C-terminal lid domain to mediate co-chaperon binding.

Heat shock proteins (HSPs) are a family of highly homologous molecular chaperons that can protect cells from damage triggered by diverse physical and chemical stresses, for example, elevation or fall of temperature, ultraviolet irradiation, medical treatment, and pathogen invasion. The expression of HSPs is precisely regulated under stress to limit the detrimental consequences and facilitate cell recovery. The HSP70 family consists of molecular chaperons of approximately 70 kDa in size that are highly conserved in all organisms (Radons, 2016). All the HSP70 chaperons share similar domain structures, including an N-terminal ATP-binding domain (NBD), a substrate-binding domain (SBD), and a C-terminal lid domain (Figure 1), and their chaperon activity relies on the hydrolysis of ATP. In the ATP-bound stage, HSP70s bind to the substrates with relatively low affinity. The affinity increases significantly after hydrolyzing ATP into ADP with the help of accessory proteins from the HSP40 family. Finally, nucleotide exchange factors facilitate the dissociation of ADP to reset the cycle (Rauch et al., 2016). In the large HSP70 family, the heat shock cognate 71-kDa protein (HSC70) is constitutively expressed and plays a major role in protein quality control, such as assisting the refolding of misfolded proteins, regulating protein translocation (Sheffield et al., 1990), and targeting protein to lysosomes or ubiquitin/proteasome machinery for degradation (Terlecky et al., 1992; Ohba, 1994). Besides these diverse cellular functions, HSC70 is widely reported to be involved in regulating the life cycle of various viruses, such as mediating attachment and endocytosis (Isa et al., 2004), penetration and uncoating, transcription and replication (Du et al., 2011), and assembly and budding (Prange et al., 1999). More importantly, HSC70 is closely related to virus-induced host immune response through presenting antigenic peptides by MHC-II to CD4^+^T cells (Auger et al., 1996) and acting as a modulator of autophagy (Beere, 2004). Due to the extensive roles of HSC70 playing in the virus life cycle, it has been studied to act as the antiviral target to inhibit virus infection. Therefore, elucidating the mechanism of HSC70 involved in viral infections will redefine our understanding of the interaction between virus and host and might contribute to developing creative antivirals especially for the treatment of emerging and resurging viruses.

### HSC70 Participates in Viral Entry

Attachment, the first crucial step to initiate infection, depends on the interaction between virus attachment proteins and cellular receptors. Exploring virus-bound cell-surface molecules has always been the hot topic of virology. HSC70 usually distributes in the cytoplasm and shuttles between cytoplasm and nucleus to participate in many biological processes. However, the exit of HSC70 from nucleus to cytoplasm is inhibited upon stress, limiting its function to the nuclear compartment (Kodiha et al., 2005). Meanwhile, studies have reported that HSC70 is also a membrane-anchored protein (Arispe et al., 2002), and it has been proved to be expressed on the cytoplasmic membrane of several different cell lines, such as MA104, Caco-2, Hep2, BHK (Guerrero et al., 2002), C6/36 (Paingankar et al., 2010), B cells (Mayer, 2005), and the suckling mouse intestinal epithelial cells (Guerrero and Moreno, 2012), and participates in virus invasion as receptor or co-receptor.

Dengue virus (DENV), a notorious insect-borne virus, has been reported to interact with several molecules to enter host cells, such as DC-SIGN in dendritic cells (Navarro-Sanchez et al., 2003), GRP78 (BiP) in HepG2 cells (Upanan et al., 2008), and β3 integrin in HMEC-1 cells (Zhang et al., 2007). HSC70 has been reported to be associated with the cell entry step of DENV through interacting with envelope glycoprotein E. HSC70 is highly expressed on the surface of C6/36 cells infected with DENV via relocation, and incubating with antibodies to HSC70 significantly blocks DENV binding to the C6/36 surface and further inhibits virus infection (Vega-Almeida et al., 2013). Apart from being a binding receptor for DENV, HSC70 also participates in rotavirus invasion. It is already known that the entry of rotavirus into epithelial cells is a multistep process that involves at least three interactions between virus and cellular receptors, including sialic acids, gangliosides, integrins, and HSC70 (Arias et al., 2002). Although different rotavirus strains enter cells through different endocytic pathways, rotaviruses, either resistant/sensitive to neuraminidase or dependent/independent on integrin, require the involvement of HSC70, and pretreating MA104 cells with antibodies against HSC70 can inhibit rotavirus infection (Gutierrez et al., 2010). To be infectious, rotavirus spike protein VP4 is cleaved into VP5 and VP8 subunits to promote virus entry into the cytoplasm (Ruiz et al., 1994). Two distinct cell surface-binding domains are present on VP5: one is the integrin-binding DGE motif located at amino acids from 308 to 310, and the other is the HSC70-binding motif located at amino acids from 642 to 658. A synthetic analog containing amino acids 642–658 blocks rotavirus infectivity but not binding to the surface of MA104 cells, indicating that HSC70 serves as a post-attachment cell receptor for the rotavirus (Zárate et al., 2003). Further study reveals that the peptide-binding domain of HSC70 involves the interaction with VP5, but its ATP-binding domain exerts a negative effect on rotavirus infection. In the presence of ATP, the infectivity of purified rotavirus to MA104 cells reduces 60% when incubated with soluble HSC70, indicating that ATPase activity of HSC70 partially inactivates rotavirus infectivity (Pérez-Vargas et al., 2006). With the growing understanding of the interconversion between bound and free conformations of HSC70, a rational assumption is that a large number of ATP in the system might constrain allosteric change of HSC70 and weaken the interaction between HSC70 and VP5, resulting in the decline of virus infectivity consequently.

### HSC70 Involves in Viral Intracellular Trafficking and Disassembly

After binding cellular receptors, viruses must devise strategies to cross the membrane barrier and uncoat capsid to expose nucleic acid for gene expression. Many enveloped viruses are internalized through clathrin-mediated endocytosis (Matlin et al., 1982). After viral attachment, adaptor protein complex 2 (AP2) concentrates cellular receptors and recruits clathrin to the designated membrane. Clathrin assembles into triskelion and induces an inward curvature with the help of coat proteins. As it gradually invaginates, the pit detaches from the membrane to form a clathrin-coated vesicle (CCV) (Brett and Traub, 2006). After detachment, clathrin is quickly removed and recycled and the vesicle goes on to fuse with the endosome. Then, viruses evolve at least three characterized mechanisms, by low pH, by receptor binding plus low pH and by receptor binding plus the action of a protease, to trigger fusion in the endosome and release viral genome (White and Whittaker, 2016). During transport among cellular compartments, HSC70 drives the clathrin assembly–disassembly cycle through providing energy (Chang et al., 2002; Xing et al., 2010). With the help of J domain proteins, such as auxilin, HSC70 binds to clathrin and dislodges triskelion in a reaction with ATP hydrolysis (Rothnie et al., 2011). In cells overexpressing ATP-binding domain-deficient HSC70 mutants, the uncoating of CCV is inhibited and cytosolic clathrin gathers with AP1 and AP2 to form an empty “cage” without receptor accumulation, indicating that HSC70 broadly regulates clathrin dynamics throughout the CCV cycle (Newmyer and Schmid, 2001). Since HSC70 contributes to the disassociation of clathrin from the vesicle coat, it undoubtedly involves the nucleocapsid disassembly of many enveloped viruses. Japanese encephalitis virus (JEV) initiates infection through clathrin-mediated endocytosis (Nawa, 1998; Yang et al., 2013). In HSC70-knockdown C6/36 cells, however, JEV only binds onto but cannot be taken up by cells to form acidified endosomes, let alone release viral RNA for further translation on the endoplasmic reticulum (ER) membrane (Ren et al., 2007; Chuang et al., 2015), suggesting that HSC70 participates in JEV infection through affecting endocytosis.

In addition to enveloped viruses, HSC70 also participates in transporting non-enveloped virus particles. Simian virus 40 (SV40) enters cells through caveolar internalization and accumulates in ER compartment (Pelkmans et al., 2001). Then, SV40 hijacks ER membrane-bound J proteins DnaJB12 (B12), DnaJB14 (B14), and DnaJC18 (C18) (Bagchi et al., 2015) to recruit SGTA (small glutamine-rich tetratricopeptide repeat-containing protein α), HSC70 (Walczak et al., 2014), HSP105 (Ravindran et al., 2015), and Bag2 (Dupzyk and Tsai, 2018) to form a cytosolic complex in a J domain-dependent manner and recruit Ubiquilin4 via a J domain-independent mechanism (Liu and Tsai, 2020), to mediate SV40 ER-to-cytosol transport. SGTA regulates the ability of HSC70 to directly interact with membrane-embedded SV40, while HSP105 and Bag2 trigger SV40 disassociate from HSC70, thereby enabling the virus to translocate across the ER membrane. Apart from assisting SV40 transport, HSC70 contributes to the export of virus ribonucleoprotein complex (vRNP) through competing with NS2 to bind to M1, indicating important roles of HSC70 in influenza virus replication (Watanabe et al., 2014).

Besides participating in virus intracellular trafficking, HSC70 associates with several virus uncoatings. After being trafficked to endosomes, reovirus goes through stepwise disassembly to expose the core for transcription. Studies have shown that HSC70 contributes to the removal of the δ fragment, cleaved from capsid protein μ1 (Bodkin et al., 1989), in an ATP-dependent manner, and further releases the transcriptionally active core into the cytoplasm. Blocking HSC70 through specific antibodies significantly inhibits δ release and, complementing with purified HSC70, totally restores the δ-release activity (Ivanovic et al., 2007), suggesting that HSC70 plays central roles in reovirus disassembly. Apart from animal viruses, HSC70 has been reported to be associated with cucumber necrosis virus (CNV) particles and incubation of recombinant HSC70-2 with CNV results in conformational changes or partial disassembly of virus capsid and produces higher numbers of local lesions on *Chenopodium quinoa*, suggesting that HSC70 plays an important role in plant virus infection (Alam and Rochon, 2017).

### HSC70 Regulates Viral Genome Replication

Viruses are small intracellular parasites and thus rely heavily on host machinery to successfully replicate their genome. Several studies have reported that HSC70 facilitates virus replication by interacting with viral protein or viral genome. Murine latency-associated nuclear antigen (mLANA) is a conserved protein of murine gammaherpesvirus 68 (MHV68), which is important for latency maintenance and acute viral replication (Virgin et al., 1997). In MHV68-infected 3T12 fibroblasts, mLANA directly interacts with HSC70 and recruits it to accumulate in the nucleus, which contribute to the formation of viral replication complexes and thereby promote viral DNA replication, the expression of late viral proteins, and ultimately virus lytic infection (Salinas et al., 2015). Duck hepatitis B virus (DHBV), a small DNA-containing virus that replicates via an RNA intermediate, has been reported to depend on HSP90 for the recognition of RNA packaging signal (ε) by viral reverse transcriptase (RT), in order to initiate replication and assemble the nucleocapsid (Hu and Seeger, 1996; Hu et al., 1997). Interestingly, the viral RT can be activated efficiently by just HSC70 and HSP40, without the need of HSP90 or other cofactors, in an *in vitro* reconstitution system (Beck and Nassal, 2003). Likewise, HSC70, especially amino acids 511–536, has been confirmed to be a supportive factor for human HBV replication, and downregulated HSC70 expression in HepG2.2.15 cells impaired HBV DNA replication by over 60% (Wang et al., 2010). Therefore, many studies have taken HSC70 as a therapeutic target against HBV, either by transfecting siRNA (Bian et al., 2012) or via treatment with chemical drugs (Du et al., 2011; Gao et al., 2011; Wang et al., 2011) to suppress HSC70 expression. Enterovirus A71 (EV-71) is a positive-stranded RNA virus, and the initiation of viral protein translation is directed by the internal ribosome entry site (IRES) in 5′-UTR in a cap-independent manner (Thompson and Sarnow, 2003). HSC70 upregulates IRES activity through interacting with 2A^*pro*^ to enhance eIF4G cleavage, and the proteolytic eIF4G dramatically obstructs cellular mRNA cap-dependent translation and assists IRES-mediated translation, which significantly promotes viral protein expression in RD cells (Ohlmann et al., 1996; Dong et al., 2018).

Chaperon activity is not limited to protein–protein interactions, and HSC70 can favor virus replication through binding regulator non-coding RNA (ncRNA). Studies have reported that many viruses, such as human immunodeficiency virus (HIV) (Sun and Rossi, 2011), DENV (Hussain and Asgari, 2014), and West Nile virus (WNV) (Mazhar et al., 2011), encode microRNA-like ncRNA to regulate virus replication. Similarly, rabies virus (RABV) transcribed a small ncRNA, called leader RNA (leRNA), to inhibit virus replication in SK-N-SH cells through interfering with the interaction between genomic RNA and the nucleoprotein. However, the expression level of HSC70 during RABV infection correlates negatively with leRNA but positively with viral genomic RNA. Further data reveals that HSC70 downregulates leRNA through interacting with its 51–59 nucleotides to promote RABV replication (Zhang et al., 2017). The Ebola virus (EBOV) genome is flanked by the 3’ leader and 5′ trailer non-coding regions (NCRs), and both the NCRs play crucial roles in regulating viral replication, transcription, and progeny genome packaging. HSC70 interacts with three motifs at nucleotide positions 26–30, 620–624, and 669–673 of trailer sequence to form a trailer-to-leader panhandle structure and further promotes EBOV 3E-5E-GFP minigenome replication in HEK293T cells (Joanna et al., 2016).

Besides positive regulation, HSC70 also exerts a negative effect on virus replication. HSC70/HSP90 has already been confirmed to be a driving force for the RNA-induced silencing complex (RISC) assembly pathway through providing ATP to load small RNA duplexes into Argonaute proteins (Iwasaki et al., 2010; Ye et al., 2012). Downregulating HSC70 in Huh7 cells suppresses miRNA-mediated host RNAi response and promotes the accumulation of DENV genomic RNA, suggesting a positive role of HSC70 in restricting DENV replication. However, NS3, an RNAi suppressor protein encoded by DENV, inhibits the loading of miRNAs into Ago1 through interacting with HSC70 to displace TRBP and finally favors virus replication in HEK293T cells (Kakumani et al., 2015).

### HSC70 Associates With Viral Morphogenesis

After biosynthesis, viruses usually recruit several host factors to promote assembly and budding. Immunogold labeling experiments suggest that HSC70 presents on the surface of hepatitis C virus (HCV) particles through interacting with the HPD (His-Pro-Arg) motif on the E2 envelope protein of the virus. In Huh7.5 cells, HSC70, HCV core, and E2 proteins were found to colocalize at the periphery of lipid droplets, an important site for HCV assembly and release. Knockdown of HSC70 with RNA interference reduces the volume of lipid droplets and inhibits viral RNA release, without influence on virus intracellular replication level, suggesting that HSC70 might modulate HCV infectivity through contributing to assembly or budding (Parent et al., 2009). The followed research demonstrates that IMB-DM122, an inhibitor of HSC70, without activity against HCV RNA polymerase or protease and not toxic to liver cells, significantly reduces J6/JFH infectivity through interfering with the encapsidation of HSC70 (Peng et al., 2010). Moreover, the allosteric HSC70 inhibitors block intracellular assembly, but not entry, replication, or translation, of infectious *Renilla* reporter JFH-1 HCV virus (Khachatoorian et al., 2016). For HBV, HSC70 not only contributes to the activation of RT but also significantly affects viral assembly. The large (L) envelop protein with its preS domain plays pivotal roles in the HBV life cycle, which largely depends on its dual topology. L protein mediates viral attachment with the preS domain translocated into the post-ER lumen (e-preS) (Neurath et al., 1986); however, it is closely related to the viral morphogenesis with the preS domain on the cytosolic side of the ER membrane (i-preS) (Ueda et al., 1991). Amino acids from 70 to 94 of the L protein, named cytosolic anchorage determinant (CAD), prevents cotranslational preS translocation and contributes to the formation of i-preS. CAD deletion relieves the suppression of preS translocation and yields a uniform topology of L protein in COS-7 cells. HSC70 selectively binds to CAD and might assist in virion formation through stabilizing the cytosolic configuration of preS (i-preS) and facilitating the contacts between L protein and viral nucleocapsid (Prange et al., 1999). The non-enveloped human papillomavirus (HPV) consists of two capsid proteins, L1 and L2, and L2 facilitates virus assembly by recruiting L1 to nuclear substructures, named PML (promyelocytic leukemia) bodies (Swindle, 1999). HSC70 interacts with the L2 C-terminus in the cytoplasm, and the complex then translocates to PML in COS-7 cells. The depletion of HSC70 blocks nuclear relocation of L2 and negatively regulates virus assembly, suggesting the indispensable role of HSC70 in integrating L2 into the viral capsid (Florin et al., 2004).

### HSC70 Correlates With Virus-Induced Host-Protective Immune Response

Chaperone-mediated autophagy (CMA) is a type of autophagy responsible for selective degradation of cytosolic proteins bearing a certain consensus amino acid motif (KFERQ) (Dice, 1990). Being a decisive component of CMA, HSC70 ensures the selectivity of target proteins and delivers the substrates to the receptor lysosome-associated membrane protein 2A (LAMP2A), in order to initiate the degradation (Kaushik and Cuervo, 2018). HCV infection induces lipid droplet accumulation and directly causes hepatocellular steatosis. Long-term co-culture of free fatty acids (FFAs) and HCV in Huh7.5 cells activates CMA and downregulates IFNAR1, impairing the IFN-α-induced JAK-STAT antiviral signal pathway (Gunduz et al., 2012). In HCV-infected Huh7.5 cells with FFA treatment, IFNAR1 is selectively degraded through interacting with HSC70 and LAMP2A on the lysosome membrane, suggesting that HSC70 inhibits host anti-HCV immunity through mediating autophagy (Ramazan et al., 2015). Another study suggests that HCV NS5A protein interacts with HSC70 and recruits HSC70 to hepatocyte nuclear factor 1 alpha (HNF-α), thereby promoting the CMA-dependent lysosomal degradation of HNF-α and facilitating virus pathogenesis (Matsui et al., 2018). Interestingly, HSC70 can directly participate in cellular host innate immunity by the DNA-dependent protein kinase (DNA-PK) DNA sensing pathway, which induces a robust and broad antiviral response via a non-classical DNA sensing pathway. Invasive viral DNA activates DNA-PK and further triggers phosphorylation of HSC70 on Ser^638^ and IRF3 on Ser^386^ to regulate downstream IFN expression. E1A oncogene of human adenovirus 5 restricts host antiviral response through antagonizing HSC70 and IRF3 phosphorylation (Burleigh et al., 2020). Besides being involved in innate immunity, HSC70 also has a role in host adaptive immunity through antigen processing and presentation. HSC70 presents peptide antigens to CD4^+^T cells, with a potential to regulate T and B cell activation and the final secretion of antibodies by plasma cells (Dengjel et al., 2005; Deffit and Blum, 2015). HSV-2 could block transporter associated with antigen processing (TAP) function in infected cells to interfere with viral peptide presentation by MHC-I to CD8^+^ T cells and induces immune evasion (Laing et al., 2010). However, HSC70-based HSV-2 peptide vaccine elicits robust CD4^+^ and CD8^+^ T cell response with good safety profile, indicating that HSC70 might facilitate CD4^+^ T cell to recognize the antigen and boost host antiviral immune, and the research finding has been applied to the phase I clinical trial (Wald et al., 2011).

## Discussion

**FIGURE 2:**
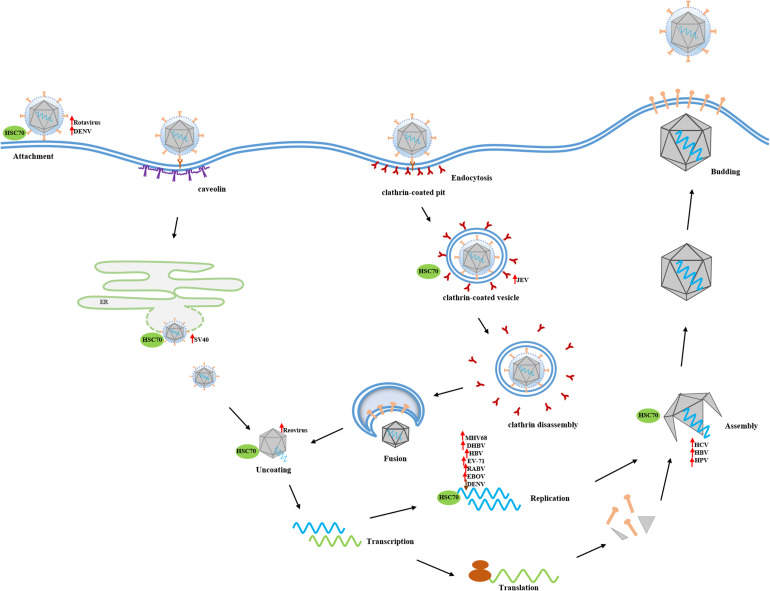
The broad spectrum of HSC70 functions in virus infections. As a multitask chaperon protein, HSC70 is hijacked to be involved in many decisive aspects of virus infection. Membrane-anchored HSC70 acts as binding receptor or post-attachment receptor to facilitate the entry of DENV and rotavirus into host cells, respectively. After crossing the membrane barrier, HSC70 contributes to viral disassembly through uncoating clathrin from CCV or assisting in releasing capsid protein in an ATP-dependent manner. Subsequently, HSC70 positively or negatively participates in the replication stage of some viruses, such as MHV68, DHBV, HBV, EV-71, RABV, EBOV, and DENV, through interacting with viral proteins or viral genome. Finally, HSC70 promotes viral morphogenesis generally via interacting with capsid protein. The up arrow means HSC70 positively regulates the viral infection stage, and conversely, down arrow means HSC70 exerts a negative effect on the viral infection stage.

As a multitask chaperone protein, HSC70 acts as a main or sub-steersman, being involved in many different cellular biological processes. With virus infection, however, these physiological functions are interrupted and HSC70 is hijacked to assist virus propagation. Many studies have demonstrated that HSC70 is a major target utilized by either enveloped or non-enveloped DNA or RNA virus to participate in different infective stages. However, HSC70 is not only coerced into facilitating virus infection but also acts as a resister to eliminate virus by boosting cellular antiviral innate and adaptive immune response. Therefore, this review summarizes the diverse roles of HSC70 in virus infections (Figure 2), to deepen the understanding of the interaction between virus and host and promote the further study of viral pathogenesis.

HSC70 is an important housekeeping protein, mostly responsible for maintaining protein homeostasis in non-stressed conditions and involved in rapidly inducible cell protection following stress situations. In contrast, HSC70 generally promotes, but rarely inhibits, virus infection through interacting with viral protein or viral gene, suggesting that many viruses benefit from host-defensive response to facilitate self-replication and it might be a potential antiviral target to limit virus infections. Based on the fact that constitutive deletion of HSC70 is embryonically lethal in mice (Cazale et al., 2009) and knockdown with siRNA induces cell death (Wang et al., 2017), allosteric regulation of HSC70 by compound or inhibitor is a better option to perturb its function. Studies have shown that the tylophorine analog, such as DCB-3503 and *rac*-cryptopleurine, specifically binds to the NBD of HSC70 and stimulates ATP hydrolysis in the presence of the poly U/UC motif of HCV RNA and thus inhibits viral translation consequently (Wang et al., 2018). As an ATP analog, adenosine derivative compound VER-155008 acts as a competitive inhibitor to bind the HSP70 family and induces conformational changes of HSC70, making it impossible for HSC70 to interact with infectious bursal disease virus (IBDV) VP2 protein and eventually inhibiting virus replication (Chen et al., 2020). Therefore, HSC70 is a promising target for antivirals and needs to be further studied.

Moreover, since HSC70 assists in the release and recycle of clathrin to form the endosome, preparing for membrane fusion, and transporting exogenous cargos to the subcellular sites, it is no doubt to play an integral role in virus invasion. It is widely believed that coronaviruses enter the host cells via two routes, the particularly important endocytic pathway and the non-endosomal pathway (Yang and Shen, 2020). However, whether HSC70 participates in coronavirus infection through influencing cell entry is still unknown.

Although being initially characterized as a chaperon to stabilize protein homeostasis, the multifunctional roles of HSC70 in cellular biological processes make it possible to participate in different stages of virus life cycle. As our understanding deepens, the underlying molecular mechanism of HSC70-regulating virus pathogenesis will be gradually elucidated, and the related antiviral drugs will be further developed.

## Author Contributions

ZW and JW searched references and wrote the manuscript. YL, XY, JZ, and YC contributed to revision of the manuscript. All authors contributed to the article and approved the submitted version.

## Conflict of Interest

The authors declare that the research was conducted in the absence of any commercial or financial relationships that could be construed as a potential conflict of interest.
